# Upregulation of Supplementary Motor Area Activation with fMRI Neurofeedback during Motor Imagery

**DOI:** 10.1523/ENEURO.0377-18.2020

**Published:** 2021-01-22

**Authors:** Salim Al-Wasity, Stefan Vogt, Aleksandra Vuckovic, Frank E. Pollick

**Affiliations:** 1 School of Psychology, University of Glasgow, Glasgow G12 8QB, United Kingdom; 2 School of Engineering, University of Glasgow, Glasgow G12 8QB, United Kingdom; 3 College of Engineering, University of Wasit, Wasit 52001, Iraq; 4 Department of Psychology, Lancaster University, Lancaster LA1 4YF, United Kingdom

**Keywords:** neurofeedback, fMRI, motor imagery, supplementary motor area

## Abstract

Functional magnetic resonance imaging (fMRI) neurofeedback (NF) is a promising tool to study the relationship between behavior and brain activity. It enables people to self-regulate their brain signal. Here, we applied fMRI NF to train healthy participants to increase activity in their supplementary motor area (SMA) during a motor imagery (MI) task of complex body movements while they received a continuous visual feedback signal. This signal represented the activity of participants’ localized SMA regions in the NF group and a prerecorded signal in the control group (sham feedback). In the NF group only, results showed a gradual increase in SMA-related activity across runs. This upregulation was largely restricted to the SMA, while other regions of the motor network showed no, or only marginal NF effects. In addition, we found behavioral changes, i.e., shorter reaction times in a Go/No-go task after the NF training only. These results suggest that NF can assist participants to develop greater control over a specifically targeted motor region involved in motor skill learning. The results contribute to a better understanding of the underlying mechanisms of SMA NF based on MI with a direct implication for rehabilitation of motor dysfunctions.

## Significance Statement

Participants in the neurofeedback (NF) group specifically learned to upregulate their supplementary motor area (SMA) functional magnetic resonance imaging (fMRI) blood oxygen level-dependent (BOLD) signal. This effect was largely restricted to the BOLD signal of the SMA. The NF was also associated with improvements in motor reaction times.

## Introduction

We investigate whether healthy participants could increase their blood oxygen level-dependent (BOLD) signal in the supplementary motor area (SMA) with the use of real-time functional magnetic resonance imaging (fMRI) neurofeedback (NF), and whether measures of motor performance would track such changes in brain activity. Previous research addressing this question (Hampson et al., 2011; Scharnowski et al., 2015; Sepulveda et al., 2016) provided mixed results and have not used an experimental design that compares performance of a true NF group to a sham NF group. In this experiment, participants were instructed to use motor imagery (MI) to increase a “thermometer” representing SMA activity.

MI is a form of motor simulation (Vogt et al., 2013) in the absence of overt movement (Blefari et al., 2015). MI and motor execution (EXE) are thought to share similar neural networks (Jeannerod, 2001), and MI plays an important role in motor learning (Gentili et al., 2010; Schuster et al., 2011). Further examination using activation likelihood estimation (ALE) analyses highlights that MI activates a large number of primary and secondary motor areas including the premotor cortex (PMC), primary motor cortex (M1), SMA, inferior frontal gyrus, precentral gyrus, middle frontal gyrus, anterior insula, inferior/superior parietal lobule (IPL/SPL), putamen, thalamus, and cerebellum (Hétu et al., 2013; Hardwick et al., 2018).

NF provides a closed loop system where a participant’s brain activity is measured and presented back to them as either a visual or an auditory feedback signal. This signal facilitates a participant’s ability to modulate their own brain activity with the aim of improving function. Previous studies using electroencephalography (EEG)-based NF have shown that healthy participants and patients can be trained to alter their scalp electrical activity in a wide range of applications such as improving cognitive functions using MI (Scherer et al., 2015; for review, see Marzbani et al., 2016). However, limitations of EEG-NF include low spatial resolution and difficulty in providing feedback from subcortical brain areas. An alternative method of NF is provided by fMRI, which measures BOLD levels and enables feedback signals from brain activity of deeper brain structures and with higher spatial resolution, albeit with lower intrinsic temporal resolution.

Several fMRI NF studies have demonstrated that participants can be trained to regulate the fMRI BOLD signal (henceforth referred to as activity) of different brain regions, such as regions responsible for emotions (anterior insula and amygdala; Caria et al., 2010; Zotev et al., 2011; Veit et al., 2012), the auditory cortex (Haller et al., 2010), language areas (Rota et al., 2009), and the visual cortex (Scharnowski et al., 2012). These studies have reported behavioral changes following NF training. Furthermore, several other NF studies have examined motor and motor-associated cortices, focusing on how NF provided during EXE (Neyedli et al., 2018) or MI (Yoo et al., 2008; Auer et al., 2015; Scharnowski et al., 2015) can enhance motor performance. Clinically, NF from sensorimotor-targeted regions can be used in motor rehabilitation related to stroke and neurologic disorders (Subramanian et al., 2011; Sitaram et al., 2012; DeCharms et al., 2005; Linden and Turner, 2016). In addition, real-time fMRI studies have shown that NF-based MI training can alter the functional connectivity between target regions and other brain regions (Marins et al., 2015; Xie et al., 2015), but the related mechanisms and link to improved motor performance is unclear.

For modulating motor cortex activity, fMRI-NF studies have used different motor regions to derive a feedback signal, including the PMC (Sitaram et al., 2012; Zhao et al., 2013; Hui et al., 2014; Marins et al., 2015), M1 (Yoo et al., 2008; Berman et al., 2012; Chiew et al., 2012; Blefari et al., 2015; Neyedli et al., 2018), and the SMA (Hampson et al., 2011; Scharnowski et al., 2015; Sepulveda et al., 2016). Specifically, fMRI-NF studies targeting the SMA have revealed mixed findings: Scharnowski et al. (2015) and Sepulveda et al. (2016) found that participants were able to increase their SMA activity during the NF training, but the lack of control groups makes these results difficult to interpret. In addition, Hampson et al. (2011) did not find a significant increase in SMA activity, possibly because of the limited number of runs used.

Given these shortcomings in the existing research, in the present study we investigated (1) whether healthy participants are able to increase the activation levels in their SMA during MI of complex actions when receiving SMA NF, and whether brain regions other than the SMA were activated during the NF; (2) to contrast the brain networks activated during real and sham NF using whole-brains analyses; and (3) whether successful SMA-NF translates to changes in behavioral measures. In contrast to the fMRI-NF studies reviewed above (Hampson et al., 2011; Scharnowski et al., 2015; Sepulveda et al., 2016), we improved the study design to include both a genuine NF group and a control group that received sham NF. An assessment of motor function was performed on all participants before and after training. If participants are able to successfully and selectively modulate SMA activity while performing a MI task, we should see improved motor function performance in the NF group only.

## Materials and Methods

### Participants

Twenty healthy participants with normal or corrected-to normal vision were recruited. Seventeen of them were right-handed and one was ambidextrous with a laterality index of 33.3 according to the Edinburgh Inventory (Oldfield, 1971). Participants were randomly assigned to two groups: 10 participants to the NF group (five males, mean age: 26.1 ± 5.1 years) who received true feedback, and 10 to the control group (seven males, mean age: 23.2 ± 2.6 years) who received sham feedback. Participants were not informed to which group they were assigned. As apparent from Table 1, there were no systematic group differences regarding age, education, and handedness score. In addition, no systematic differences were found on the Vividness of Movement Imagery Questionnaire-2 (VMIQ-2; Callow and Roberts, 2010). The ethics committee of College of Science and Engineering approved this study. All participants provided their informed consent for the experiment.

**Table 1:** Demographic features for participants in the NF and control groups

		NF group (mean ± SD)	Control group (mean ± SD)	*p* value (two-tailed *t* test)
Age (years)		26.1 ± 5.1	23.2 ± 2.6	0.175
Education (years)		17.2 ± 2.3	16.6 ± 2	0.621
Handedness		81.4 ± 15.7	74.3 ± 23.7	0.490
MI vividness	Third person perspective	21.6 ± 10.1	18.6 ± 4.8	0.462
	First person perspective	18.5 ± 4.2	18.1 ± 4.3	0.839

### Imaging parameters and fMRI NF platform

The study was performed on a 3T Siemens Tim Trio MRI scanner at the University of Glasgow Centre for Cognitive Neuroimaging (CCNi) with a 32-channel head coil. T1-weighted structural scans were acquired at the beginning of the experiment (TR = 2300 ms, TE = 2.96 ms, 192 sagittal slices, 1-mm^3^ isotropic voxels and image resolution 256 × 256). T2*-weighted functional scans were collected with an echoplanar imaging (EPI) sequence (TR = 2000 ms, TE = 30 ms, whole-brain coverage with 32 axial slices, 0.3-mm gap and 3-mm^3^ isotropic voxel).

The NF system used Turbo-BrainVoyager version 3.2 (Brain Innovation) and a custom script running on MATLAB (MathWorks Inc.) to visualize the feedback signal as a thermometer. An LCD projector displayed the thermometer onto a rear projection screen that could be viewed through a mirror mounted on the head coil.

### Experimental procedure

All participants underwent the same procedure, which consisted of a questionnaire interview outside the scanner, a prescan behavioral test, a localizer run, fMRI NF training (true feedback for the NF group and sham feedback for the control group), and a postscan behavioral test.

### Behavioral test

We used a Go/No-go task to assess motor performance. In this task, a response must be given in the “go” trials and inhibited in the “no-go” trials, providing a cognitively engaging scenario. It has been shown that there is activation in the SMA during go trials (Liddle et al., 2001). Participants completed 250 trials of this task before and after the NF training session, this is task was repeated for each hand separately. They were instructed to press the space bar of a conventional keyboard using their index finger as quickly and accurately as possible when a go-trial was displayed (green target), and to inhibit their response (that is, to keep the index finger positioned above the space bar) when a no-go trial was presented (blue target). The task was run using Inquisit 5 software. Each trial consisted of a fixation point (+) presented for 800 ms, followed by a blank white screen for 500 ms, followed by a rectangular cue (horizontal 2.5 × 7.5 cm, or vertical 7.5 × 2.5 cm, where stimulus orientation was not informative) that was displayed for one of five intervals (100, 200, 300, 400, 500 ms) to reduce the temporal warning effect. Finally, go and no-go targets were colored green and blue, respectively, and were presented for 1000 ms or until a response occurred (Fillmore et al., 2006).

A three-way mixed effect ANOVA (hand × group × pre/post) was performed to analyze between and within group effects. A paired-sample *t* test was used as a *post hoc* test to compare between the pre-post experiment reaction time of each group and hand separately.

### Functional localizer

**Figure 1.**
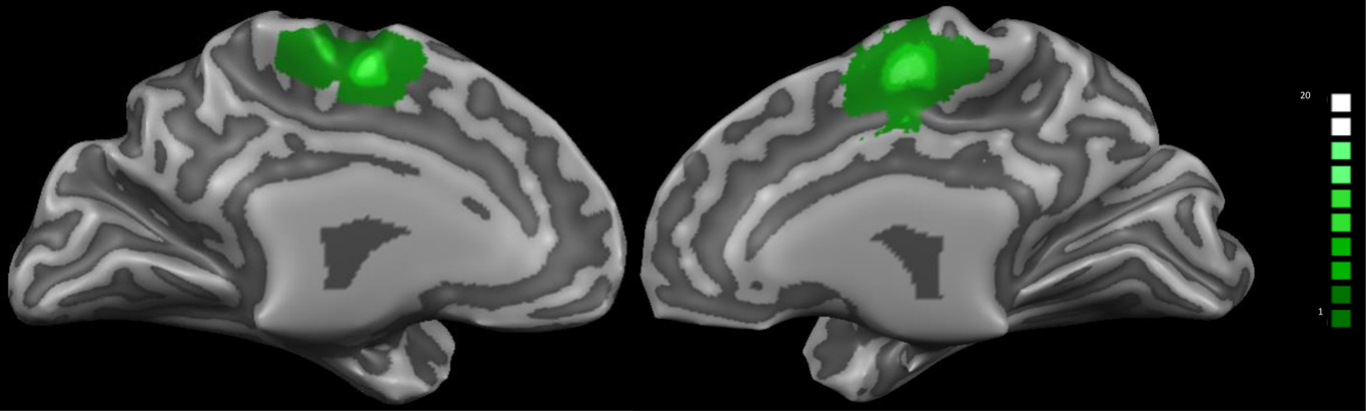
Overlap of individual SMA-ROI for the 20 participants of both groups. The subject-specific SMA-ROIs were identified before the NF training using a functional localizer run during an index finger tapping task.

The NF training session started with a functional localizer run, to identify the SMA, from which the participant received the feedback signal. The localizer lasted for ∼5 min and consisted of seven fixation blocks (16 s) interleaved by six blocks of bimanual index finger-tapping (30 s). Written instructions were given to the participants to either “rest” or “tap.” The functional data were preprocessed and analyzed online with an accumulative general linear model (GLM) embedded in Turbo-BrainVoyager. The SMA-region of interest (ROI) was delineated from the active voxels (threshold of *t* > 5.0) within a rectangle that was positioned anterior to the precentral sulcus and superior to the cingulate sulcus, as shown in Figure 1. The ROIs were defined in each participant’s native space and subsequently used for the NF training runs to derive the NF signal. For further analysis, we normalized these ROIs into Talairach space, as illustrated in Table 2, and identified them based on the nearest gray matter using the Talairach Daemon (Lancaster et al., 2000).

**Table 2:** Subject-specific SMA-ROI in Talairach space

	Subject number	Anatomical area	Talairach coordinates			Number of voxels
			*x*	*y*	*z*	
NF group	1	LH, medial frontal gyrus	−6	−7	52	1163
	2	LH, medial frontal gyrus	−6	−19	58	702
	3	LH, medial frontal gyrus	−3	−10	52	1754
	4	RH, medial frontal gyrus	6	−10	58	1333
	5	LH, medial frontal gyrus	−4	−14	48	1463
	6	LH, medial frontal gyrus	0	−7	49	1520
	7	LH, paracentral lobule	−9	−25	52	2984
	8	RH, medial frontal gyrus	9	−10	47	1730
	9	RH, medial frontal gyrus	3	−10	52	2569
	10	RH, medial frontal gyrus	9	−13	52	1186
Control group	11	RH, medial frontal gyrus	2	−11	51	1683
	12	LH, medial frontal gyrus	−10	−8	48	1520
	13	LH, medial frontal gyrus	−7	−17	51	1539
	14	LH, medial frontal gyrus	−4	−5	57	1344
	15	LH, cingulate gyrus	−10	−11	45	1408
	16	LH, medial frontal gyrus	−7	−5	57	2086
	17	LH, medial frontal gyrus	−4	−8	57	1792
	18	RH, Cingulate Gyrus	8	−2	48	2072
	19	RH, medial frontal gyrus	8	−8	54	1848
	20	LH, medial frontal gyrus	−4	−10	49	1268

### fMRI NF

**Figure 2.**
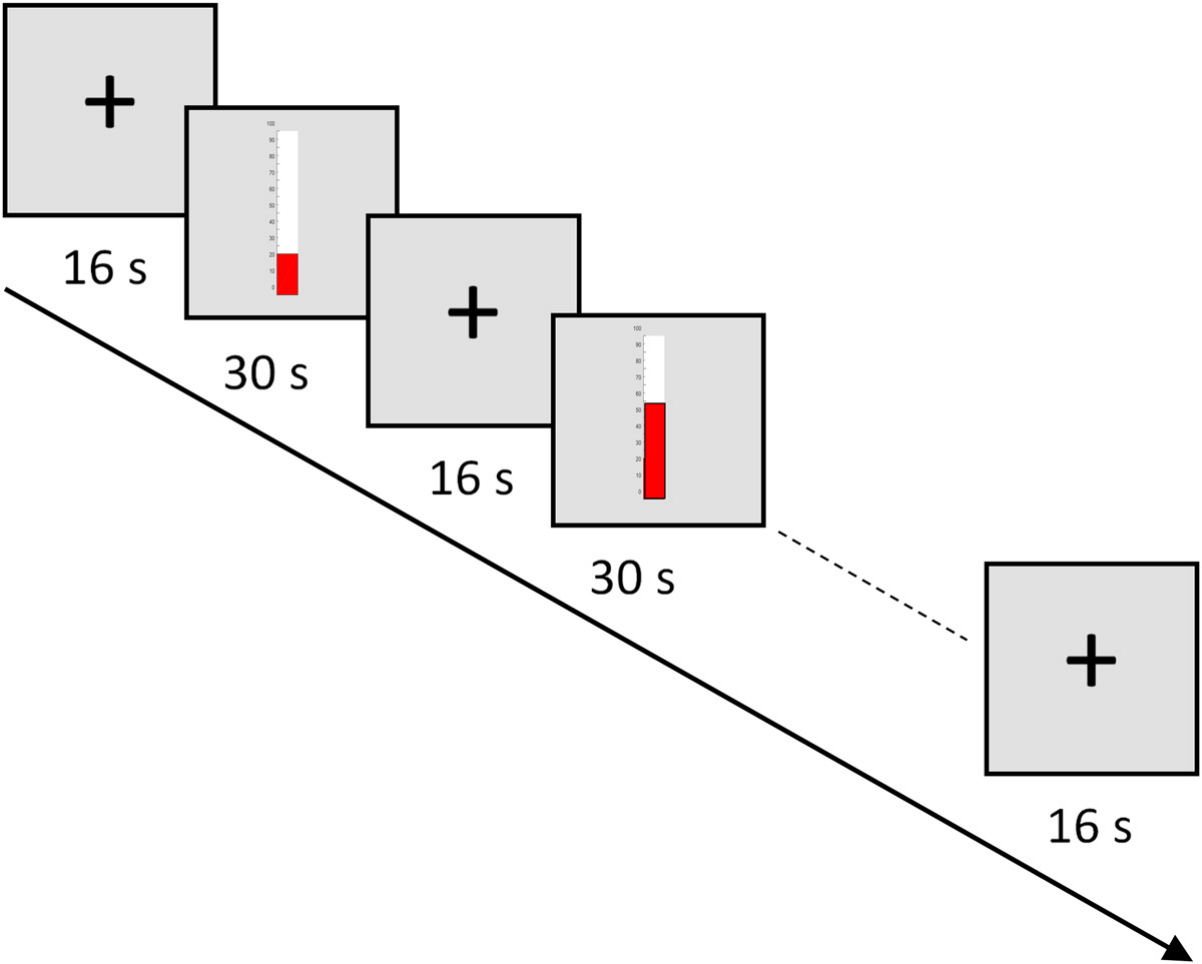
fMRI NF training paradigm of one run. A run lasted for 430 s and consisted of nine 30-s-long NF blocks alternating with ten 16-s-long fixation (rest) blocks.

All participants took part in seven 430-s-long NF training runs, where they were instructed to upregulate their targeted ROI by engaging in a MI task of complex body actions of their choice. Each NF training run consisted of nine 30-s-long blocks of NF interleaved with ten 16-s-long fixation blocks, as shown in Figure 2. During the NF blocks, participants saw a thermometer, and were instructed to increase its level by imagining their own execution of complex actions. During the fixation blocks, participants looked at a fixation cross and were instructed to relax and count upwards “1,2,3…” to keep their baseline signal low. Engaging in more complex mathematical operations has been shown to activate motor-related networks (Hanakawa, 2011; Berman et al., 2012).

The control group was presented with sham feedback that was randomly chosen from individual prerecorded signals across seven participants in the experimental group (yoked feedback; Chiew et al., 2012; Hui et al., 2014).

### Online data analysis

Real-time fMRI data analysis and NF presentation was performed using Turbo-BrainVoyager software and MATLAB. The scanner transmitted the acquired fMRI data volume by volume to the analysis computer that hosted Turbo-BrainVoyager through a network connection. Functional data were preprocessed in real time, which included linear de-trending, slice timing correction, 3D motion correction and spatial smoothing using a Gaussian kernel with full width at half maximum (FWHM) of 8 mm, then added to a cumulative GLM.

The feedback signal consisted of a thermometer with a continuously updated red column height at each TR of 2000 ms, based on the following equation:

$$Column\;height(t)=\left(\frac{ROI_{SMA}(t)-ROI_{SMA\_base}}{ROI_{SMA\_base}}\right)-\left(\frac{ROI_{reference}(t)-ROI_{r\_base}}{ROI_{r\_base}}\right).$$

Where *ROI_SMA_(t)* and ROI*_reference_(t)* are the average BOLD signals of the SMA-ROI and a reference ROI during the NF block at time *t*. *ROI_SMA_base_* and *ROI_r_base_* are the average BOLD signals of the last three volumes in the fixation block of SMA-ROI and reference ROI, respectively. The reference ROI, used to correct for global scanning effects, encompassed a rectangular region covering all the voxels within an axial slice (*z* = 10) distant from the motor network, and showed no activation when the localizer run was analyzed.

### Offline data analyses

The raw data were preprocessed offline using BrainVoyager QX 2.8.4 (Brain Innovation). The first two volumes of each run were discarded to allow for T1 equilibration effects. The preprocessing of the remaining functional data involved slice scan-time correction with cubic-spline interpolation, 3D motion correction with Trilinear/Sinc interpolation, linear trend removal, high-pass filtering with a cutoff set to three cycles and spatial smoothing with 4-mm FWHM isotropic Gaussian kernel. All functional images of each subject were aligned to the first functional volume after the anatomic scan and spatially normalized to Talairach space to enable group analysis across participants (Talairach and Tournoux, 1988).

In the first level analysis, all preprocessed functional data of each subject were analyzed using a GLM with two predictors (tapping and rest for the localizer, feedback and rest for NF), convolved with a hemodynamic response function. Covariates derived from six head motion parameters (Johnston et al., 2010; Van Dijk et al., 2012), an estimate of the white matter signal (Jo et al., 2010; Zilverstand et al., 2015), and the ventricular signal (Birn et al., 2009; Zilverstand et al., 2015) for modeling physiological artefacts (e.g., respiration and cardiac effects) and scanner instability.

#### ROI analysis

To examine the NF training success, β weights were estimated using a ROI-GLM analysis for each NF run of each subject’s ROI for the SMA (identified by the functional localizer presented in Table 2) and were used as an indicator for the NF success. This was assessed via a two-factorial (group × run) repeated-measure ANOVA, as well as via paired *t* tests between the first and the last run in each group. Furthermore, a linear regression of the average β weights over NF runs was used to examine the upregulation over runs as an index of self-learning. In addition, an event-related average time course was computed for the last and first NF runs.

Similarly, the β weights of six additional regions of the motor network (bilateral M1, PMC, and PPC), that were delineated using RFX-GLM analyses of the NF and localizers runs across the two groups, were estimated to assess the influence of modulating the SMA activity during the NF training on this wider network. Statistically this was tested via two-factorial (group × run) ANOVAs for each ROI, as well as via linear regressions of the average β weights of each ROI. In addition, we contrasted the NF effects on the SMA against the effects on the additional regions directly in a three-factorial contrast analysis (group × ROI × run).

#### Whole-brain analyses

Group data were evaluated based on a second level random effect analysis GLM (RFX-GLM). The obtained statistical maps were corrected for multiple comparisons using cluster-level thresholding (Goebel et al., 2006). In this method, the uncorrected voxel-level threshold maps were submitted to a whole-brain correction criterion based on the estimate of the map’s spatial smoothness and on an iterative procedure (Monte Carlo simulation) for estimating cluster-level false-positive rates. After 1000 iterations, the minimum cluster-size that produced a cluster-level false positive rate (α) of 5% was applied to threshold the statistical maps.

A first whole-brain RFX-GLM analysis was performed for the localizer runs. The contrast “tapping versus rest” was computed and a threshold was set at *p *<* *0.01, with a cluster-level thresholding of 899 mm^3^. Activations were mostly found in motor-related areas, however the SMA was not included here, most likely because of between-subjects variability .

In addition, a whole-brain second level RFX-GLM analysis was conducted for the NF runs for each group separately (*p *<* *0.01, with cluster-level thresholding of 981 mm^3^ for the NF group and 1139 mm^3^ for the control group). A two-sample *t* test was performed to directly contrast NF and control groups, thresholding at *p *<* *0.01 with a cluster-level thresholding of 432 mm^3^. For examining the interaction between run and group, we also ran a voxel-wise two-way mixed ANOVA with the factors run (seven runs, within subjects) and group (two groups, between subjects). The interaction effect of the whole-brain ANOVA maps was thresholded at *p *<* *0.01 uncorrected, with a cluster-level threshold of 1242 mm^3^.

## Results

### Behavioral results

**Figure 3.**
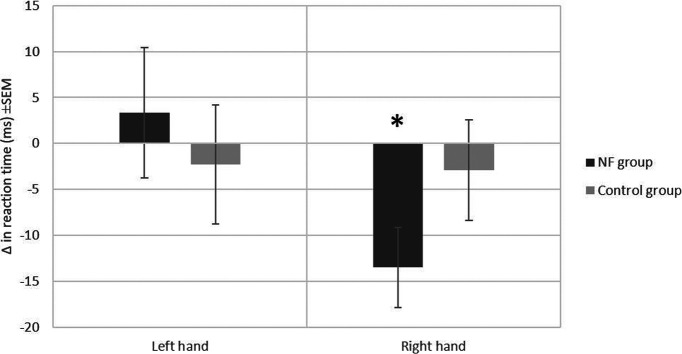
Reaction time (ms) differences before and after the self-regulation of both hands for the two groups. Errors bar represent the SEM; *p = 0.013.

Figure 3 shows the difference in reaction time of the two groups before and after the NF training for both hands. The repeated measures ANOVA of the reaction times showed a significant interaction effect of hand × pre/post-test, *F*_(1,18)_ = 6.1, *p* = 0.02, and a significant hand × group × pre/post-test interaction, *F*_(1,18)_ = 5.2, *p* = 0.03. No significant effects were found for hand (*F*_(1,18)_ = 0.06, *p* = 0.8), group (*F*_(1,18)_ = 0.99, *p* = 0.33), or pre/post-test (*F*_(1,18)_ = 1.02, *p* = 0.326), nor for the hand × group interaction (*F*_(1,18)_ = 2.6, *p* = 0.12), or the group × pre/post-test interaction (*F*_(1,18)_ = 0.1, *p* = 0.74). Paired-sample *t* tests between pre/post-test reaction times, run separately for each group and hand, revealed a significant effect of NF training in the right hand of the NF group (*t*_(9)_ = 3.106, *p* = 0.013) but not in the control group (*t*_(9)_ = 0.535, *p* = 0.606). There was no significant effect for the left hand in either group (NF group: *t*_(9)_ = 0.471, *p* = 0.648; control group: *t*_(9)_ = 0.353, *p* = 0.732).

### ROI analyses

**Figure 4.**
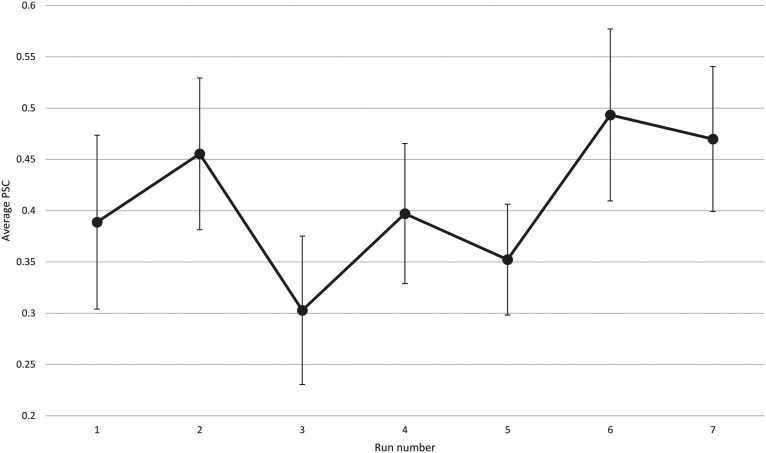
The average PSC of the NF group calculated according to Equation 1. Error bar indicates SEM.

**Figure 5.**
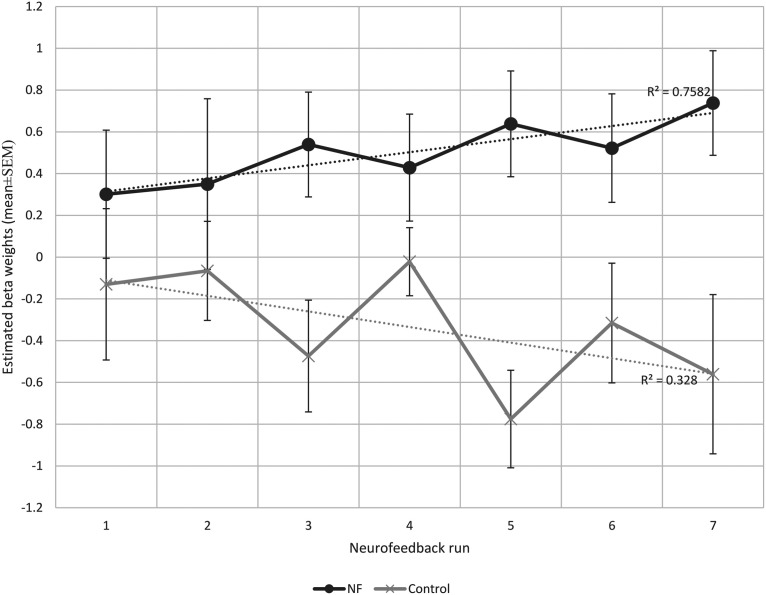
The mean β weights of NF and control groups across runs. The β weights were used as an indicator of the success of self-regulation. For statistics see text.

Each participant completed seven NF runs in one session. Participants of the NF group learned to increase the brain activity acquired from their functionally localized SMA regions as shown in Figure 4. Most participants reported that they used MI of bimanual hand punching or boxing. The average β weights in the SMA estimated off-line during each run of the NF and control group are shown in Figure 5. The two-way mixed effects ANOVA of the β weights indicated a significant main effect of group (*F*_(1,18)_ = 40.7, *p *<* *0.0001), while the main effect of run was not significant (*F*_(1,18)_ = 0.18, *p *=* *0.98). More importantly, when testing for a linear trend for run, we found a near-significant effect for the group × run interaction (*F*_(1,18)_ = 4.2, *p *=* *0.053). Subsequent paired *t* tests revealed a significant increase in SMA activity from the first to the last run (*t*_(9)_ = −1.83, *p *<* *0.04) in the NF group, whereas the control group showed no significant change (*t*_(9)_ = 0.88, *p *<* *0.2).

In addition, a linear regression highlighted a gradual increase in the mean SMA activity across runs in the NF group indicating a learning effect (y = 0.062×+0.252, *F*_(1,5)_ = 15.68, *r*^2^ = 0.75, *p *<* *0.01). The control group did not show such learning progress (y = −0.074×–0.035, *F*_(1,5)_ = 2.44, *r*^2^ = 0.32, *p *=* *0.17). The difference between slopes was significant, *t*_(10)_ = 2.73, *p *=* *0.02.

**Figure 6.**
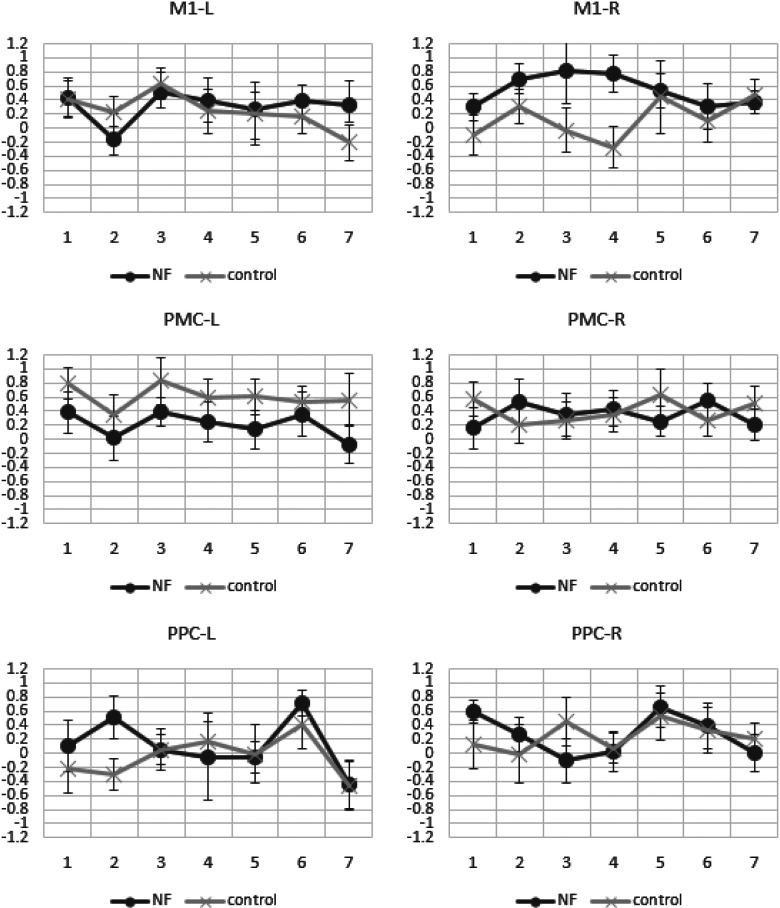
The mean β weights of NF (black line) and control (gray line) groups across NF runs of six frontoparietal motor regions. M1, PMC, posterior parietal cortex (PPC), left (L), right (R), vertical axis: mean β weights, horizontal axis: run number, the error bars represent the standard error of the mean.

In contrast to the clear trend for a differential effect of the NF training on the SMA, such effects were either less pronounced or absent in the six other regions of the motor network analyzed here, namely bilateral M1, PMC, and PPC as shown in Figure 6. That is, in the two-way ANOVAs for these ROIs, none of the group × run interactions was significant (for bilateral M1: *F*s < 2.4, *p*s* *>* *0.13; for bilateral PMC and PPC: *F*s < 0.63, *p*s* *>* *0.43). In line with these results, the regression analyses did not show significant increases/decreases in the mean activity across runs of both groups for these ROIs as summarized in Table 3.

**Table 3:** The linear regression of the six additional frontoparietal regions

Cortical area	*x*	*y*	*z*	Group	Regression	*F*_(1,5)_	*p*	*R*^2^
LH, M1	−33	−15	47	NF	y = 0.019×+0.229	0.19	0.67	0.03
				Control	y = −0.084×+0.578	5.69	0.06	0.53
RH, M1	25	−25	40	NF	y = −0.03×+0.66	0.49	0.5	0.09
				Control	y = 0.62×–0.11	1.44	0.28	0.22
LH, PMC	−33	−4	46	NF	y = −0.03×+0.66	0.9	0.38	0.15
				Control	y = −0.2×+0.69	0.44	0.53	0.08
RH, PMC	27	−10	46	NF	y = 0.002×+0.34	0.005	0.94	0.001
				Control	y = 0.009×+0.36	0.074	0.79	0.01
LH, PPC	−34	−34	25	NF	y = −0.048×+0.31	0.4	0.55	0.07
				Control	y = 0.21×–0.13	0.13	0.73	0.02
RH, PPC	−46	−46	40	NF	y = −0.02×+0.37	0.19	0.67	0.03
				Control	y = 0.034×+0.1	0.77	0.41	0.13

No significant increase/decrease of the estimated β weights shown across runs of both groups.

The specificity of the modulatory effects of the NF training for the SMA was examined further in a three-factorial contrast analysis (group × run × ROI) where each ROI was contrasted against the mean of the remaining ROIs (using the Deviation contrast in SPSS, and linear trends for run). Importantly, this analysis indicated that the group × run effect was significantly more pronounced in the SMA than in the remaining ROIs, *F*_(6,18)_ = 6.1, *p *=* *0.024. Note that this contrast analysis also indicated a marginally significant second order interaction for the left M1, *F*_(6,18)_ = 4.5, *p *=* *0.046. However, in contrast to the results for the SMA, the two-factorial ANOVA for the left M1 carried a non-significant group × run interaction, *F*_(6,18)_ = 2.4, *p *=* *0.13, as reported above, which compromises the interpretation of the second order interaction for this region. In summary, the effect of the NF training on the BOLD signal was largely restricted to the SMA, while among six other regions of the motor network, only the left M1 showed a similar, but statistically not significant effect.

**Figure 7.**
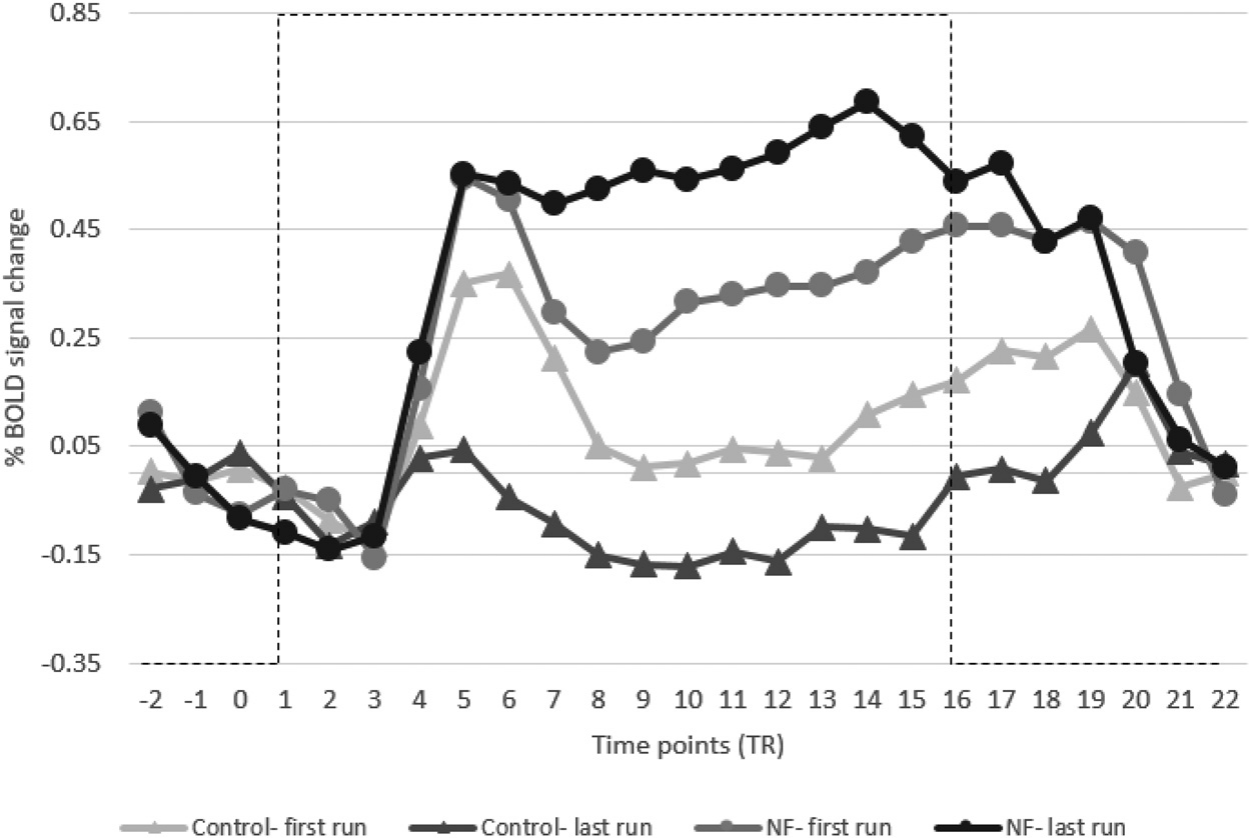
Average BOLD signal change of target SMA regions of NF and control groups comparing the first and last runs. NF training helped to increase the SMA activity of the NF group (black lines) compared with the control group where it decreased it (gray lines). Error bars are SEM. Dashed lines represent the task block.

Additionally, Figure 7 shows the averaged time course of the BOLD signal during the NF blocks of both groups. This figure plots the first and the last runs for both groups and shows an increase in SMA activity for the NF group.

### Whole-brain analyses of NF runs

**Figure 8.**
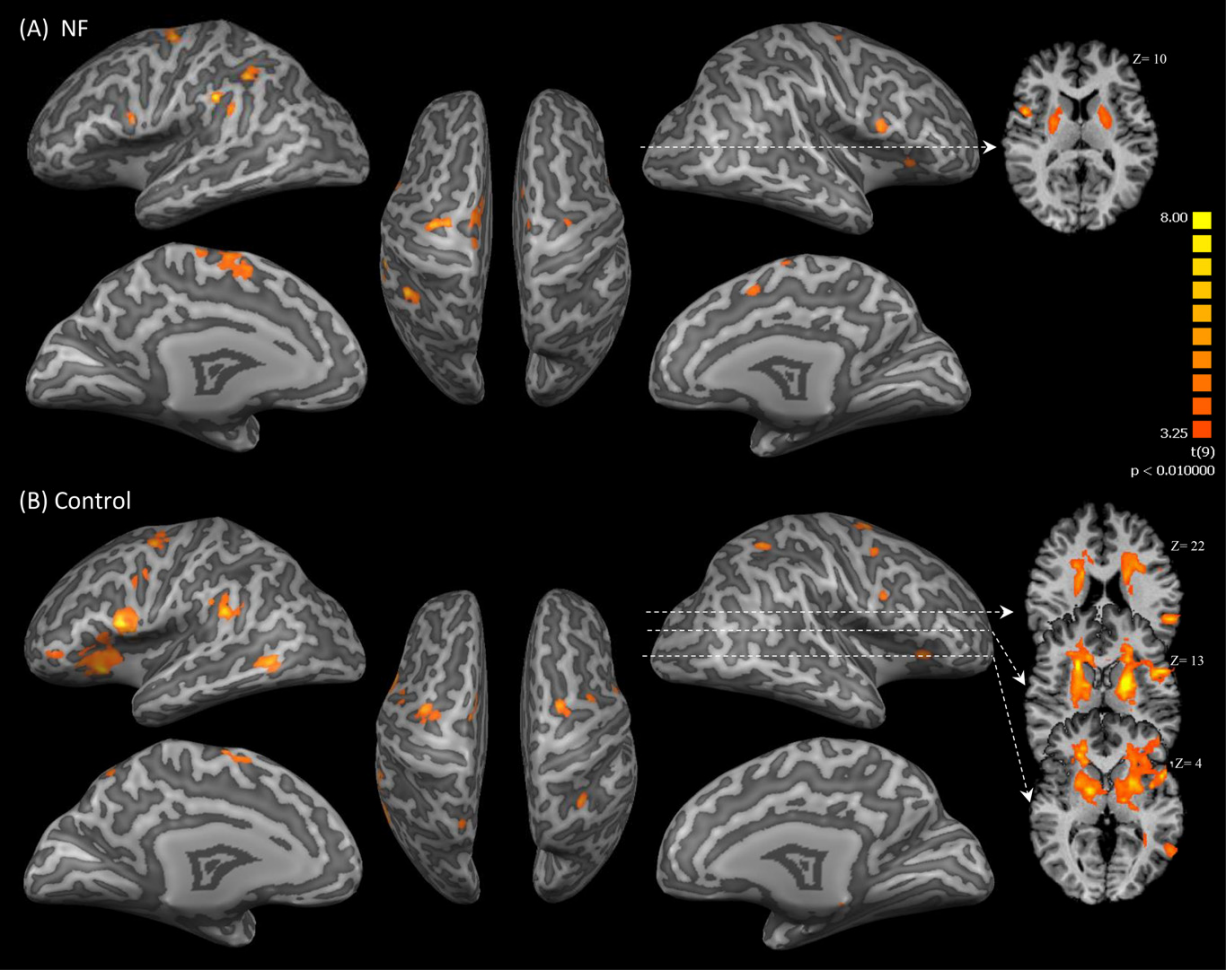
Results of the RFX-GLM analysis of NF runs shown for the (***A***) NF group and (***B***) control group. These activations are significant at *p* < 0.01 (cluster size >981 and >1139 mm^3^, respectively).

For overview, a whole-brain RFX-GLM analysis was performed across runs for both NF and control groups as illustrated in Figure 8 and listed in Table 4. For the NF group, activations were found in the left SMA, IPL, and bilateral precentral gyrus (left PMC and right Broca’s area) and in the basal ganglia. For the control group the bilateral basal ganglia, bilateral middle frontal gyrus, left IPL, and left middle temporal gyrus were found activated.

**Table 4:** Clusters of brain activation for NF and control groups

Group	Cortical area	x	y	z	t	p value	Size
NF	LH, lateral globus pallidus	−21	−7	4	5.2415	0.00053	1924
	LH, IPL, BA 40	−60	−28	34	7.9406	0.00002	1258
	LH, supramarginal gyrus, BA 40	−42	−40	37	7.7510	0.00002	1263
	LH, precentral gyrus, BA 6	−30	−13	52	7.2034	0.00005	4863
	RH, putamen	24	−1	7	5.8323	0.00024	1995
	RH, precentral gyrus, BA 44	48	5	10	7.1174	0.00005	1405
Control	LH, middle temporal gyrus, BA 21	−57	−55	4	7.2142	0.00005	1504
	LH, putamen	−18	−1	13	12.8867	0.00001	24743
	LH, IPL, BA 40	−57	−34	22	7.5089	0.00003	1756
	RH, caudate body	21	17	13	11.4746	0.00001	15864
	RH, middle frontal gyrus, BA 6	36	−4	46	6.7628	0.00008	1605

*x*, *y*, *z* are given in Talairach coordinates. LH, left hemisphere; RH, right hemisphere; BA, Brodmann area.

**Figure 9.**
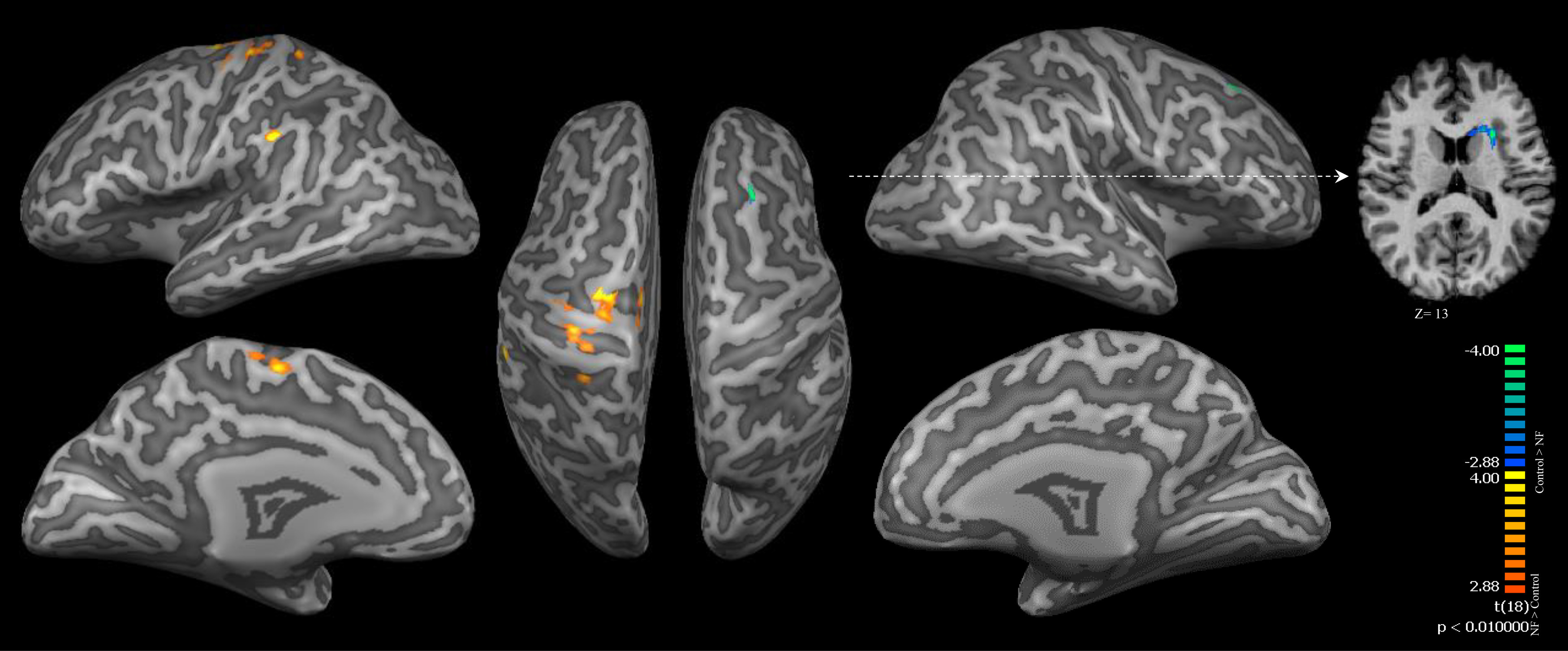
A contrast map between the RFX-GLM of NF and control groups. Red/yellow color represents significant actions in the NF group while the blue/green color indicates higher activation in the control group. The map was thresholded at *p* < 0.01 (cluster size >432 mm^3^).

In addition, a two-sample *t* test was performed to contrast the RFX-GLM maps of both groups directly. The NF group showed higher activations in clusters located in the left sensorimotor cortex (SMA, M1, and primary sensory cortex) compared with the control group that showed higher activations in the left claustrum and right middle frontal gyrus, as illustrated in Figure 9 and listed in Table 5.

**Table 5:** Comparison of brain activations between NF and control groups

	Cortical area	x	y	z	t	p value	Size
NF > control	LH, medial frontal gyrus, BA 6	0	−9	49	4.2104	0.00052	875
	LH, precentral gyrus, BA 6	−33	−7	58	5.9098	0.00001	1994
Control > NF	RH, middle frontal gyrus, BA 8	36	26	43	−6.1933	0.00001	2628
	LH, claustrum	−24	14	13	−4.9600	0.00010	1120

*x*, *y*, *z* are the Talairach coordinated. LH, left hemisphere; RH, right hemisphere; BA, Brodmann area.

**Figure 10.**
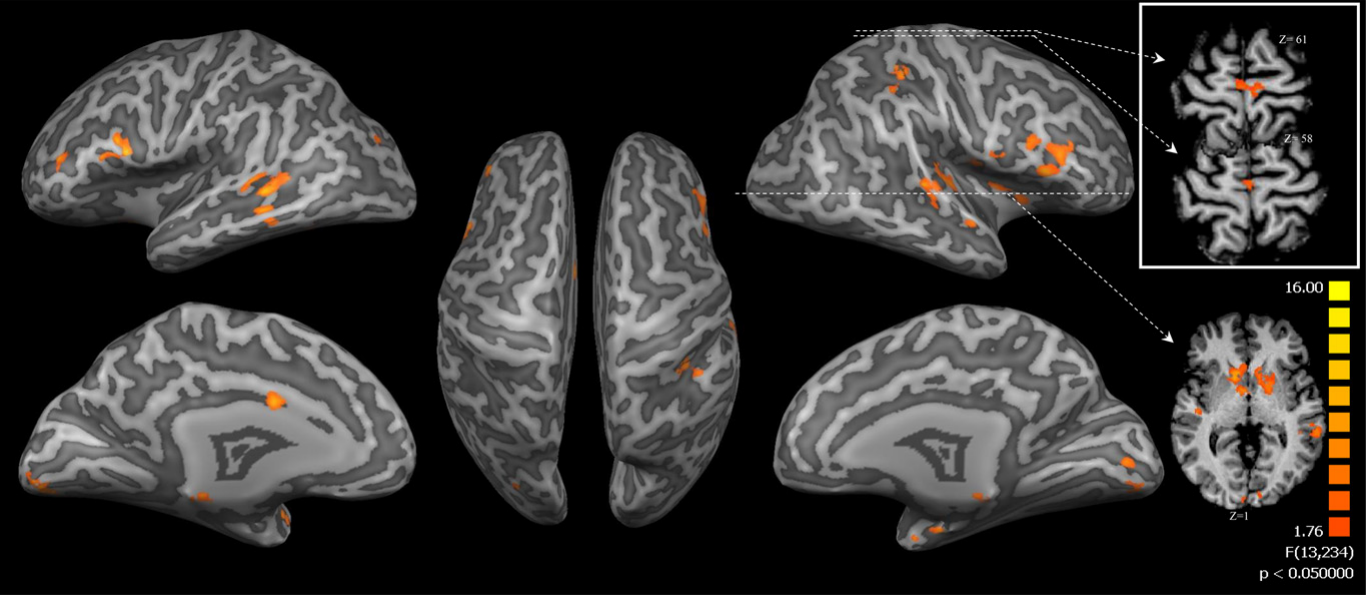
Two-factorial ANOVA examining the interaction (group × run) effect. The brain slabs (in the white rectangular) show uncorrected activation in the SMA regions. The surface maps were thresholded at *p* < 0.05 (cluster size >1424 mm^3^).

The interaction (groups × runs) of the whole-brain two-factorial ANOVA showed an activation of bilateral middle frontal gyrus, superior temporal gyrus, lingual gyrus, and caudate head as shown in Figure 10 and listed in Table 6. Furthermore, the same figure shows a small cluster of uncorrected activation (*p *<* *0.05) in the SMA.

**Table 6:** Clusters of brain activation for the ANOVA interactions effect

Cortical area	*x*	*y*	*z*	*t*	*p* value	Size
RH, superior temporal gyrus, BA 42	63	−28	7	3.8164	0.00001	1269
RH, precentral gyrus, BA 6	54	−1	13	3.3213	0.00019	2150
RH, middle frontal gyrus	39	22	19	4.8032	0.00001	1871
RH, caudate head	12	11	1	3.863	0.00001	3147
LH, lingual gyrus, BA 18	−9	−76	−8	4.1983	0.00001	3655
LH, lentiform nucleus	−18	2	1	2.9345	0.00053	2115
LH, precentral gyrus, BA 44	−51	10	13	3.2926	0.00016	1310
LH, superior temporal gyrus, BA 41	−45	−34	4	3.7605	0.00001	1431

*x*, *y*, *z* are the Talairach coordinates. LH. left hemisphere; RH, right hemisphere; BA, Brodmann area.

### Correlation between behavioral measures and NF performance

We examined the data for correlations between behavioral measures and NF performance in the NF group. NF performance was measured as the difference in β weights obtained from the SMA, calculated between the first and last NF runs. To check for individual differences because of MI capabilities we conducted a linear regression between VMIQ scores and NF performance. This regression produced a non-significant result (y = 1.180–0.034×, *F*_(1,8)_ = 0.94, *p *=* *0.36, *R*^2^ = 0.10), suggesting that our effect was not driven by individual differences. To check for a relationship between reaction time in the behavioral task and NF performance we calculated a linear regression between the change in reaction time between and NF performance for the NF group. This produced a non-significant result (y = −17.878 + 10.021×, *F*_(1,8)_ = 3.44, *p *=* *0.10, *R*^2^ = 0.30) indicating that the change in reaction time was not accounted for by the amount of change in BOLD activation in the SMA.

## Discussion

In this study, we demonstrated that healthy volunteers could learn, in a single session, to increase the activity in their functionally localized SMA region, during a MI task of complex body actions while receiving a continuous feedback signal (displayed as a thermometer bar). This feedback signal represented the activity of individually localized SMA regions in the NF group, whereas the control group received a sham feedback signal. In the NF group, the estimated β weights of the SMA increased with the number of runs, indicating a practice effect in modulating the SMA activation. In addition, the NF group showed faster responses in the reaction time task after the training, while no such effect was present in the control group.

The first aim of this study was to explore the ability of healthy participants to increase the SMA activity guided by NF in a single training session. Our results showed that participants of the NF group, who engaged in MI and received feedback information from their SMA region, increased their SMA activation. The β weights of the NF group progressively increased, which would reflect the gradual increase in ability to self-regulate. In contrast to the NF group, participants of the control group, who received a yoked feedback signal, did not increase their SMA activity (or the estimated β weights). This lack of increase resulted presumably because the provided feedback signal did not correspond to the changes in their targeted brain regions and thus did not reinforce the relationship between brain activity and feedback signal. A complete understanding of the neural mechanisms by which self-regulation is obtained is an unresolved theoretical problem in the field of NF (Sitaram et al., 2017). Sitaram et al. (2017) proposed the possibility of two distinct neural networks to be involved in NF, one network involving cognitive factors and explicit processing of reward and another network involving more automatic aspects of reward processing. Such dual-process mechanisms can be related to the current experiment where participants were given the cognitive task of performing MI as a mean to maximize their feedback signal.

These findings of increased SMA activity guided by a single NF session confirm those of previous studies (Banca et al., 2015; Blefari et al., 2015; Scharnowski et al., 2015), which indicated that a single session of NF training is sufficient to elicit NF-related practice effects. In contrast, the additional six regions of the motor network did not show significant effects of NF, which demonstrates the specificity of NF training on modulating only the SMA activity. Participants in both groups had a comparable capability to perform MI as measured by the VMIQ-2 questionnaire. The debriefing after the scanning of the participants in NF group revealed that most of them initially struggled to identify the best imagery strategy. A number of different MI strategies during the NF training were reported, including first-person perspective MI of bimanual punching or boxing. In contrast, participants of the control group were frustrated about not being able to control the thermometer level using MI strategies. Common documented strategies in successful modulation include MI of clenching and pitching (Yoo et al., 2008; Chiew et al., 2012; Blefari et al., 2015) and sequential finger movements (Berman et al., 2012; Neyedli et al., 2018). MI and EXE have been shown to activate common cortical regions including the SMA, bilateral PMC, M1, posterior parietal lobe, and the cerebellum (Hanakawa et al., 2008; Hétu et al., 2013; Sharma and Baron, 2013). The shared neural substrate between different motor modalities supports the feasibility of NF training using MI to enhance motor performance. Finally, it is worth considering if the increase in SMA β weights might have been because of the MI instruction per se, rather than a result of the NF. Typically, neuroimaging studies on practice effects of pure MI tasks, without involving NF, show neural efficiency effects, that is, activation decreases with practice (Sakreida et al., 2018). Therefore, we consider it unlikely that the present effect can be attributed to the MI instruction alone.

Based on the above, the second aim of this study was to compare between the brain networks involved in NF training during real and sham feedback conditions. The whole-brain RFX-GLM analysis of each group separately revealed widespread brain activation beyond the targeted area (SMA). For the NF group these activations included the left SMA, PMC, IPL, and bilateral basal ganglia, and for the control group the bilateral PMC, basal ganglia, middle frontal gyrus, and right IPL. The SMA is involved in motor planning and control (Grefkes et al., 2008; Nachev et al., 2008). Indeed, the NF group showed an increase in the left SMA activation during the NF training, consistent with previous findings of left hemisphere dominance in practice-related activation increase regardless of the trained hand (Halsband and Lange, 2006). The PMC plays an important role in planning and preparation of movements (Hoshi and Tanji, 2007; Hétu et al., 2013). Our results of activation in the left PMC highlight the dominant role of this area in movement selection (Bestmann et al., 2008), while the right PMC activation are consistent with spatial processing during the early stage of motor learning (Halsband and Lange, 2006). The IPL activation could be related to the integration of visuomotor information (Halsband and Lange, 2006), or the internal recruitment of stored motor representations (Cooke et al., 2003). Particularly, the left IPL is suggested to be involved in the storing/retrieval of motor plans (Van Elk, 2014) and visually guided motor tasks (Torres et al., 2010). Further, the basal ganglia is involved in motor processes and cognitive functions, such as learning based on the assessment of outcomes (Arsalidou et al., 2013). Interestingly, the putamen is thought to be essential in the learning of novel complex motor actions and less important in well trained movements (Ceballos-Baumann, 2003), which is consistent with the pattern of basal ganglia activation observed in the NF group, which suggests that a task can be conducted using fewer neural substrates, as fast learning proceeds (Poldrack, 2000). Importantly, in contrast to the NF group, the control group showed widespread activation in the basal ganglia. This widespread activation is potentially related to processes of executive function when participants in the control group unsuccessfully attempted to adapt their MI to improve the feedback signal. This would have involved trying different MI actions and possibly modulating attention to different aspects of the imagined movement, which would be cognitively demanding. For example, Arsalidou and colleagues (2013) highlight the connection between executive function and different regions of the basal ganglia: planning that activates the head and body of the right caudate, working memory that activates the bilateral putamen, and reward processes that activate anterior parts of bilateral caudate head. Comparison of brain activation between the NF and the control groups revealed significantly higher activations in the left SMA, M1 and PMC of the NF group, further supporting our hypothesis that the NF group was able to increase the activation of SMA during NF training. The interaction (groups × runs) of the whole-brain ANOVA showed significant activation in bilateral ventrolateral prefrontal cortex and temporoparietal junction which could be related to the imagination of actions and the integration of imagery and memory by remembering the visual appearance (Zimmer, 2008), respectively. The SMA was not differentially activated in the interaction, but this could be because of the conservative analysis used (no linear trend for the main effect RUN). Given that such an interaction in the SMA would have been congruent with the obtained ROI-GLM results, future research might re-examine this point.

Finally (aim 3), we wished to test the hypothesis that successful self-regulation would be related to changes in measures of motor function. Our results were mixed, with between group differences supporting the hypothesis, while changes in motor performance of individual participants within the NF group failing to support the hypothesis. In the Go/No-go task, participants were instructed to respond as quickly and accurately as possible, and related decreases in reaction time between pre-test and post-test were indeed found in both groups for the right hand. Importantly, this decrease was only significant in the NF group. This finding further supports that MI training guided by true NF can be used to bring the brain into a state where movement vigor is enhanced. The Go/No-go task involves planning and initiation of movements during the Go trials, and inhibition of inappropriate actions during the No-go trials. These processes are likely mediated by the SMA (Nachev et al., 2008). The SMA has direct connections to M1, the ventrolateral thalamus, and to the spinal cord via the corticospinal tract (Johansen-Berg et al., 2004; Nachev et al., 2008; Arai et al., 2012), and it has been shown that modulating SMA activity can increase the cortical excitability of M1 (Arai et al., 2012; Shirota et al., 2012). Our finding of faster motor reaction times following SMA-contingent NF training is thus consistent with motor physiology. Despite the positive finding of an overall NF group decrease of reaction times and increase in β weights our hypothesis was not confirmed at the individual participant level; we did not find a significant correlation between change in reaction times and change in β weights for individuals in the NF group.

Rounding up, in line with the previous studies of fMRI NF (Bray et al., 2007; Chiew et al., 2012; Sitaram et al., 2012; Zhao et al., 2013; Scharnowski et al., 2015), we demonstrated that the use of a MI task during real-time fMRI NF is effective in upregulating activity specifically in the targeted motor region (here, SMA), and that it can improve motor performance. Our study presents the first controlled study that highlights the feasibility of increasing SMA activation during a single session. Clinically, learning control over the SMA could be used to treat Tourette’s syndrome where SMA activity is linked to motor tics (Bohlhalter et al., 2006; Hampson et al., 2011) and Parkinson’s disease where the SMA activity is reported to be underactive (Munzert et al., 2009; Subramanian et al., 2016).

In conclusion, our results demonstrate the feasibility of fMRI NF to upregulate SMA activity and illustrate the remarkable plasticity of the brain to adapt its function to novel situations. By learning to influence the height of a visually presented thermometer participants can self-modulate their SMA activity in a single session. Notably, this upregulation was largely restricted to the SMA, while other regions of the motor network did only exhibit marginal effects of the NF training. Furthermore, the sucessful regulation of SMA activity also translated into enhanced motor response times in a visuo-motor task. Although significant theoretical questions remain as to the manner in which learning of self-modulation is achieved (Emmert et al., 2016; Sitaram et al., 2017; Watanabe et al., 2017), how NF can be developed into therapeutic applications or to answer fundamental questions of brain function is a rapidly expanding area of research (Hampson et al., 2019).

## Synthesis

Reviewing Editor: Satu Palva, University of Helsinki Decisions are customarily a result of the Reviewing Editor and the peer reviewers coming together and discussing their recommendations until a consensus is reached. When revisions are invited, a fact-based synthesis statement explaining their decision and outlining what is needed to prepare a revision will be listed below. The following reviewer(s) agreed to reveal their identity: James Sulzer.

Synthesis

The authors conducted a study using fMRI neurofeedback of SMA. They compared an experimental to a yoked sham control group and found improved reaction time in the neurofeedback group. Both reviewers found your paper well written and straightforward with a well-designed experiment. Both reviewers also found some issues that should be addressed before the publication is warranted.

Comments.

- Liberal statistical thresholds were used for RFX and PPI analysis. Given the current concerns with cluster-level thresholding, please confirm that the rate of enhanced false positives is taken care of in your statistical thresholding. I’m especially concerned with a cluster level threshold of 0.05 with the PPI (See Eklund, A., Nichols, T. E., & Knutsson, H. (2016). Cluster failure: why fMRI inferences for spatial extent have inflated false-positive rates. Proceedings of the National Academy of Sciences, 201602413). Could the same results be obtained with stricter threshold?

- Please clarify the sham control condition. Was it yoked to the average of 7 people? How were the 7 people yoked exactly?

- The Fig. 5 is unclear. Was it tested if there was a significant difference between slopes in Fig 5? There seems to be a groupxrun interaction that was nearly significant, but is this what is referred to in the text.

- Was the increase in reaction time correlated to neurofeedback performance?

-Was there any correlation to VMIQ and neurofeedback performance?

-The study explored with functional connectivity analysis functional coupling between the seed region to other brain areas. Was the possibility of common input ruled out?

Minor

-"learned to up-regulate their SMA activity” -- “SMA activity” refers to fMRI signal. Please mention this.

- “This neuromodulatory effect” -- It is not entirely clear what is meant by “neuromodulatory” here. Perhaps “this up-regulation” instead.

- “and with higher spatial resolution” What about temporal resolution/delay?

- “inhibitory and activational components of motor control” I do not understand what this means. Please clarify; perhaps expand.

- The introduction misses reference to subramaniam et al. 2011. It was a pilot study on a PD patients, but should be noted.

- There are many small formatting and language issues that better proofreading would catch, e.g. “figure” capitalization, parentheses, etc.

-"Accordingly, in the present study” I do not see exact correspondence between the sentences before and after this statement. Please rephrase.

- “increased their SMA activation using MI as a strategy” This sentence reads like a strategy was discovered. Alternatively, it was instructed. Please remove ambiguity.

- “widespread activation in the basal ganglia due presumably to subjects attempting to discover the best visual MI task using trial-and-error strategies.” The need to activate basal ganglia is not clear. Perhaps, some references?

- “ The SMA has direct connections to the corticospinal tract” You probably mean that SMA connects to the spinal cord through the corticospinal tract.

- “thus increasing the SMA activity will lead to reduced motor reaction times.” Please improve this explanation.
